# Concerns Discussed on Chinese and French Social Media During the COVID-19 Lockdown: Comparative Infodemiology Study Based on Topic Modeling

**DOI:** 10.2196/23593

**Published:** 2021-04-05

**Authors:** Stéphane Schück, Pierre Foulquié, Adel Mebarki, Carole Faviez, Mickaïl Khadhar, Nathalie Texier, Sandrine Katsahian, Anita Burgun, Xiaoyi Chen

**Affiliations:** 1 Kap Code Paris France; 2 Centre de Recherche des Cordeliers INSERM Sorbonne Université, Université de Paris Paris France; 3 Unité d'Épidémiologie et de Recherche Clinique Hôpital européen Georges Pompidou Assistance Publique - Hôpitaux de Paris Paris France; 4 Département d’informatique médicale Hôpital européen Georges Pompidou Assistance Publique - Hôpitaux de Paris Paris France; 5 Département d’informatique médicale Hôpital Necker-Enfants Malades Assistance Publique - Hôpitaux de Paris Paris France; 6 Paris Artificial Intelligence Research Institute Paris France

**Keywords:** comparative analysis, content analysis, topic model, social media, COVID-19, lockdown, China, France, impact, population

## Abstract

**Background:**

During the COVID-19 pandemic, numerous countries, including China and France, have implemented lockdown measures that have been effective in controlling the epidemic. However, little is known about the impact of these measures on the population as expressed on social media from different cultural contexts.

**Objective:**

This study aims to assess and compare the evolution of the topics discussed on Chinese and French social media during the COVID-19 lockdown.

**Methods:**

We extracted posts containing COVID-19–related or lockdown-related keywords in the most commonly used microblogging social media platforms (ie, Weibo in China and Twitter in France) from 1 week before lockdown to the lifting of the lockdown. A topic model was applied independently for three periods (prelockdown, early lockdown, and mid to late lockdown) to assess the evolution of the topics discussed on Chinese and French social media.

**Results:**

A total of 6395; 23,422; and 141,643 Chinese Weibo messages, and 34,327; 119,919; and 282,965 French tweets were extracted in the prelockdown, early lockdown, and mid to late lockdown periods, respectively, in China and France. Four categories of topics were discussed in a continuously evolving way in all three periods: *epidemic news and everyday life*, *scientific information*, *public measures*, and *solidarity and encouragement*. The most represented category over all periods in both countries was *epidemic news and everyday life*. *Scientific information* was far more discussed on Weibo than in French tweets. Misinformation circulated through social media in both countries; however, it was more concerned with the virus and epidemic in China, whereas it was more concerned with the lockdown measures in France. Regarding *public measures*, more criticisms were identified in French tweets than on Weibo*.* Advantages and data privacy concerns regarding tracing apps were also addressed in French tweets. All these differences were explained by the different uses of social media, the different timelines of the epidemic, and the different cultural contexts in these two countries.

**Conclusions:**

This study is the first to compare the social media content in eastern and western countries during the unprecedented COVID-19 lockdown. Using general COVID-19–related social media data, our results describe common and different public reactions, behaviors, and concerns in China and France, even covering the topics identified in prior studies focusing on specific interests. We believe our study can help characterize country-specific public needs and appropriately address them during an outbreak.

## Introduction

Since the identification of the first cases of COVID-19 in Wuhan, China in December 2019, the epidemic has quickly spread throughout China and many other countries worldwide. In response to the rising numbers of cases and deaths, China, followed by many other countries, implemented measures to control the epidemic and preserve their health systems. China enforced the quarantine and lockdown of cities and, subsequently, whole provinces at the end of January 2020. Traffic within urban areas was restricted, and all inner-city travel was prohibited unless permitted. All entertainment venues and public places were closed; all public events were cancelled. Subsequently, the control became more stringent, and a universal and compulsory stay-at-home policy for all residents was adopted [1]. On March 13, 2020, the World Health Organization declared Europe the second epicenter. The most affected countries included Italy, Spain, France, and Germany. Measures have consisted of the closure of borders, the closure of educational institutions, the closure of museums and theaters, the closure of shops and restaurants, restrictions on movement, and the suspension of public gatherings with small groups of people [2].

Control measures have been effective in many countries. Tian et al [3] showed that the lockdown of Wuhan and the national emergency response in China were strongly associated with a delay in epidemic growth during the first 50 days of the epidemic based on statistical and mathematical analyses of the temporal and spatial variation in the number of reported cases. Kraemer et al [4] demonstrated that drastic control measures substantially mitigated the spread of COVID-19 with real-time mobility data and detailed case data, including travel history. Similarly, lockdown measures adopted in Europe dramatically reduced viral transmission in Italy [5], Germany [6], and the United Kingdom [7], resulting in large reductions in the basic reproduction number, R0, from an average of 3.8 prior to the lockdown to below 1 in many countries [8]. France had the most rapid reductions, with an R0 of approximately 0.77 when these measures were lifted [9].

Although these interventions could be implemented with the apparent adherence of the population, it is of interest to the public health community and policy makers to assess public opinions and the impact of such interventions on individuals and populations as expressed on social media [10], which may differ in different countries.

Social media has demonstrated its value for sharing experiences, opinions, and feelings, and for disseminating important public health messages and research findings in emergency situations and during pandemics [11-13]. Here, we present an effort to analyze and compare the evolution of topics discussed on social media during the COVID-19 lockdown in China and in France.

## Methods

### Social Media Data Collection

We focused on the most commonly used microblogging networks in China and France (ie, Weibo and Twitter, respectively). The Weibo data were extracted using Weibo search engine queries, which return messages containing a specified keyword for a predetermined search period. To gather a maximum amount of data, a query was made for each extraction keyword and for each day of the period of interest. Posts returned by those queries were scraped along with their metadata. Tweets were extracted using the Twitter Search application programming interface (API), which makes it possible to query tweets containing a specific set of keywords and returns the IDs of tweets. Those IDs were then used to collect the tweet content and its metadata using the same API.

 

Two categories of keywords were considered to extract messages from Chinese and French social media: (1) COVID-19–related keywords (eg, COVID or coronavirus) and (2) lockdown-related keywords (eg, lockdown or lockdown lift). A set of synonymous terms in Chinese and French were identified for each category using an iterative process. All keywords used for extraction are listed in Multimedia Appendix 1.

As global online discourse has shown rapid evolutions over time during the pandemic [14], we considered three periods related to the start and end date of the lockdown in each country to analyze the evolving topics on Chinese and French social media:

1. Prelockdown period: 1 week before the lockdown started
2. Early lockdown period: from lockdown implementation to 10 days after
3. Mid to late lockdown period: from the 11th day after lockdown implementation to the lifting of the lockdown

More specifically, the three periods are (1) January 16-22, (2) January 23 to February 2, and (3) February 3 to April 7 for Chinese Weibo, and (1) March 10-16, (2) March 17-27, and (3) March 28 to May 10 for French tweets.

### Data Preprocessing

The first step consisted of filtering posts with respect to the language. For Chinese post extraction from Weibo, all words with Latin characters were removed except the keywords (eg, severe acute respiratory syndrome); for French post extraction from Twitter, we used the Twitter Search API to filter for messages in French. Forwarded Weibo messages and retweets were removed. Time stamps and regular expressions of periods of time were tagged. Stop words were removed for both the Chinese and French messages. Stemming was performed using the Porter [15] algorithm for French tweets. Tokenization was carried out for each corpus to split the texts into smaller parts, named tokens, for topic model estimation. Finally, to reduce the sparsity of our data, we chose to keep tokens appearing at least 10 times in the whole corpus. The detailed data preprocessing steps for both languages are shown in Multimedia Appendix 2.

### Topic Model

A biterm topic model was used to identify the topics in both corpora without prior knowledge. A topic is defined as a subject of discussion, which amounts to tokens that frequently appear together in a corpus. The biterm topic model considers the whole corpus as a mixture of topics, where each co-occurring pair of tokens (the biterm) is drawn from a specific topic independently. A post is thus represented as a combination of the topics associated with each biterm in the post and at a certain proportion. Since Twitter posts are short, often composed of one sentence, we attributed only the most prominent topic to a post [16]. To maintain a homogenous method, the same restriction was applied for Weibo posts.

A biterm topic model was applied separately to each corpus of the three periods. The number of topics was empirically set to 15 for each period, which balanced the quality of topics and the feasibility of qualitative comparisons.

### Qualitative Analysis and Validation

Each topic was interpreted and labeled using the top 20 token terms (the tokens with the highest per-topic probabilities) and at least 60 randomly selected topic-specific posts. With the objective of ensuring high quality labeling, interpretation of the topics was performed by two authors independently for each corpus as follows: authors XC and CF for the Chinese topics and authors MK and CF for the French topics. The labels resulting from each pair of interpreters were integrated, and consensus was reached if necessary.

Moreover, to ensure that all topic labels were explicit, blind validation was performed. For each corpus, two authors, who were different from the interpreters (authors MK and PF for Chinese and authors PF and AM for French), were asked to determine what theme each topic was dealing with based only on the topic label. The interpreters then evaluated their statements. Topic labels that were misunderstood by both guessing authors were renamed.

All topics in both corpora for the three periods were finally summarized into broader categories to facilitate comparisons across the periods and between the two countries.

## Results

### Social Media Data Collection

A total of 6395; 23,422; and 141,643 unique Chinese Weibo messages, and 34,327; 119,919; and 282,965 unique French tweets were extracted in the prelockdown, early lockdown, and mid to late lockdown periods, respectively, in China and France. An overview of the corpora are shown in Table 1.

**Table 1:** Extraction overview.

Lockdown period	China (n=171,460)			France (n=437,211)	
	Start and end dates	Messages, n	Start and end dates		Messages, n
Prelockdown [t_0_ – 7, t_0_)^a^	Jan 16-23	6395	Mar 10-16		34,327
Early lockdown [t_0_, t_0_ + 10]^a^	Jan 23 to Feb 2	23,422	Mar 17-27		119,919
Mid to late lockdown [t_0_ + 11, t_1_)^b^	Feb 3 to Apr 7	141,643	Mar 28 to May 10		282,965

^a^t_0_ denotes the day on which the lockdown began.

^b^t_1_ denotes the day on which the lockdown was lifted.

### Topics

The topic labels, proportions, and top terms for each period and each corpus are shown in Multimedia Appendices 3-8. All topics were grouped into four categories according to the theme that they mainly addressed: *epidemic news and everyday life*, *scientific information*, *public measures*, and *solidarity and encouragement*. The first category contains topics that discuss the statistics (prevalence, mortality, etc) and other news and facts about the epidemic, which have been evolving everyday and are highly associated with personal emotions and activities. It is a similar case for the category of *public measures* (ie, people often talked about public measures and their personal opinions on these measures simultaneously). Therefore, in these two categories, we further annotated fact-related topics and more subjective ones.

### Qualitative Analysis of Topics on Chinese Social Media

**Figure 1:**
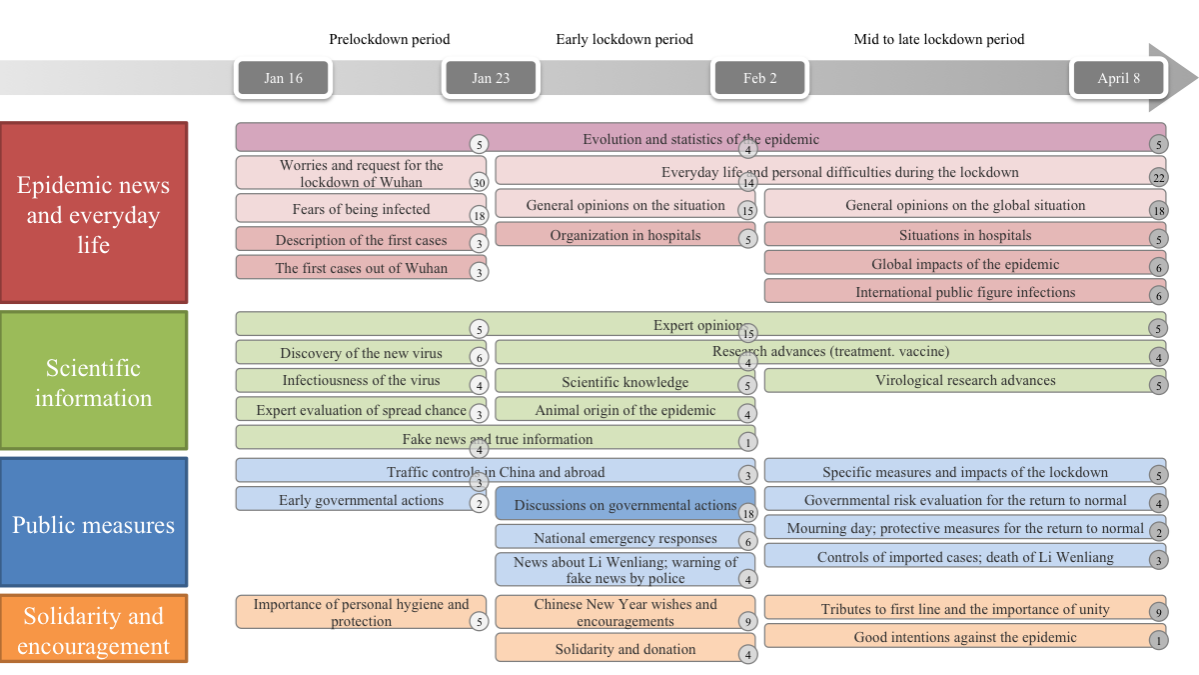
Topics identified on Weibo during the three periods. All topics are colored according to the category and aligned for the three periods. The factual topics are colored in dark red and dark blue, and the subjective topics are colored in light red and light blue for the first and the third category, respectively. The percentage of each topic is indicated in a circle at the bottom right.

The topics on Chinese social media in all three periods are shown in Figure 1. 

**Figure 2:**
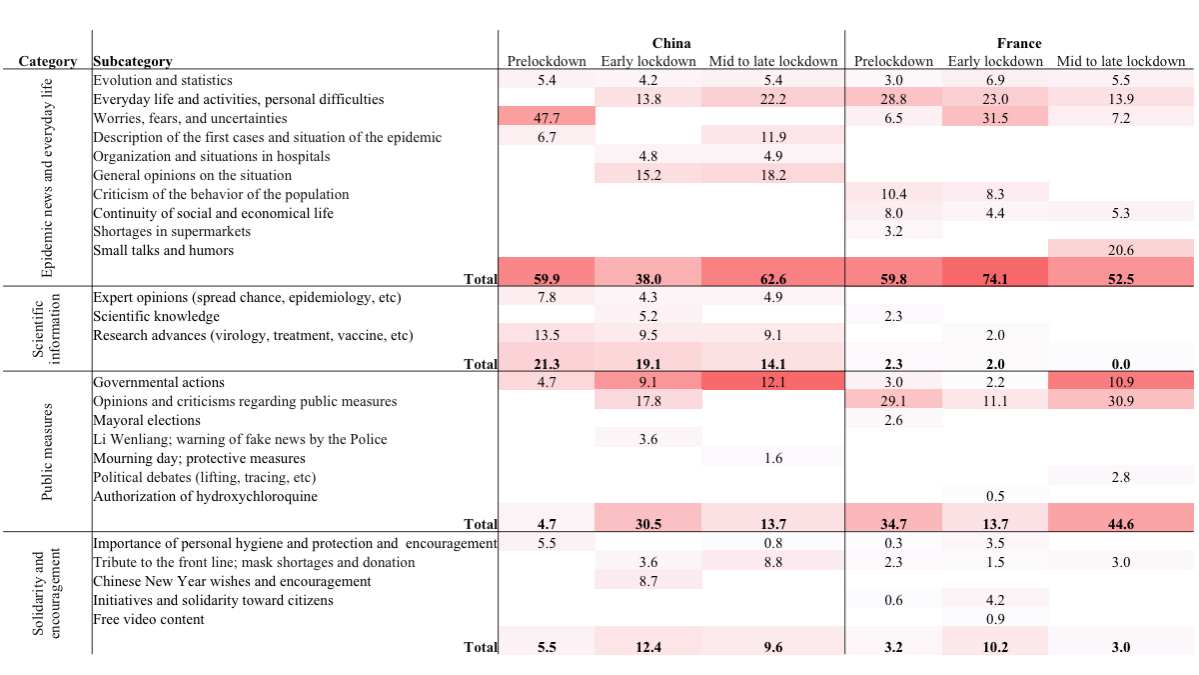
Comparison between topics on Weibo and Twitter. The percentage of messages of each category and subcategory were shown for all three periods in both countries. The color reflected the proportion.

The most represented category was *epidemic news and everyday life*, corresponding to 60% (3837/6395), 38% (8900/23,422), and 63% (89,235/14,1643) of the messages in the first, second, and third periods, respectively (Figure 2). Topics related to the evolution of the epidemic were addressed in all three periods. Before the lockdown, the Weibo messages mainly conveyed fears of being infected and worries regarding the spread of the virus, which led to requests for the lockdown of Wuhan by Weibo users and descriptions of the first cases in and outside of Wuhan. After lockdown implementation, users shared more comments and opinions on their everyday lives, the organization in hospitals, the situation of the epidemic, and its impacts in China (early lockdown period) and worldwide (mid to late lockdown period). These impacts included economic difficulties and environmental improvement.

*Scientific information* was the second most represented topic category, corresponding to 21% (1343/6395), 20% (4684/23,422), and 14% (19,830/141,643) of the messages in the first, second, and third periods, respectively. Topics related to expert opinions were present in all periods. During the prelockdown and early lockdown periods, some topics related to early knowledge about the virus (eg, infectiousness or animal origin) and topics related to the need to distinguish between fake news and true scientific information were shared. Posts like “Be wary of the following fake news:..., and the truth is that...” or “Refute a rumor:...” were often observed. Subsequently, during the second and third periods, the topics shifted to research advances in virology, treatments, and vaccines.

*Public measures* were intensively discussed in the early lockdown period (7026/23,422, 30%) versus the prelockdown (320/6395, 5%) and mid to late lockdown (19,830/141,643, 14%) periods, and the topics covered early measures such as traffic controls, emergency responses such as home quarantine, and the impact of these governmental actions. Topics related to the lifting of the lockdown were identified during mid to late lockdown (eg, evaluations of the situation by regional governments and preventive measures implemented for the return to normal). Some messages related to Li Wenliang, the Chinese ophthalmologist who issued the alert about mysterious pneumonia cases and subsequently died of COVID-19, were identified during the lockdown as part of topics related to other subjects.

We also identified topics related to *solidarity and encouragement* in all three periods, and such topics represented 5% (320/6395), 12% (2811/23,422), and 10% (14,164/141,643) of the messages in the first, second, and third periods, respectively. These topics were more related to the importance of personal hygiene, protection, and respect for measures at early stages, and to solidarity and donation, the importance of unity, and tributes to frontline workers later on. As the Chinese New Year was the second day after lockdown implementation, one topic related to New Year wishes and hope for the future was identified.

Two irrelevant topics were identified for the prelockdown period due to the polysemy of the Chinese word “隔离,” which means not only “quarantine” but also “foundation makeup” or “median barrier.” These two topics, related to makeup and traffic accidents, were excluded from our analysis.

### Qualitative Analysis of Topics on French Social Media

**Figure 3:**
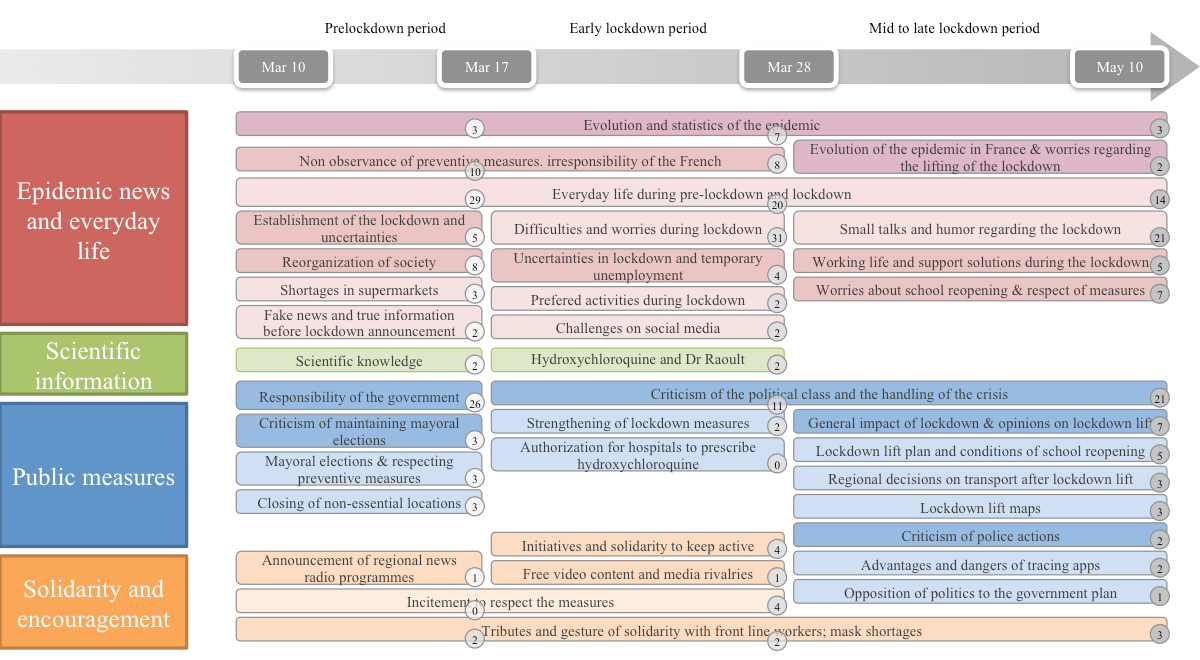
Topics identified on Twitter during the three periods. All topics are colored according to the category and aligned for the three periods. The factual topics are colored in dark red and dark blue, and the subjective topics are colored in light red and light blue for the first and the third category, respectively. The percentage of each topic is indicated in a circle at the bottom right.

The topics on French social media are shown in Figure 3. 

The most represented category was also *epidemic news and everyday life*, with 60% (20,596/34,327), 72% (86,342/119,919), and 54% (152,801/282,965) of the messages in the first, second, and third periods, respectively. The themes addressed in all three periods included the evolution and statistics of the epidemic, everyday life and activities, the continuity of social and economic life (reorganization of society after the announcement of early measures, temporary unemployment during early lockdown, and working life during mid to late lockdown), and concerns (uncertainties before the lockdown, worries and difficulties during early lockdown, and worries regarding company and school reopenings after the lifting of the lockdown). Fake news regarding the lockdown and criticisms of the lack of respect for preventive measures were identified only at early stages, whereas Twitter users started to share humoristic messages and jokes as the lockdown constraints became more familiar.

Regarding *scientific information*, one topic related to the virus and the seriousness of the situation was addressed during the prelockdown period (6865/34,327, 2%), and one topic related to the controversy surrounding the use of hydroxychloroquine in France was addressed during early lockdown (23,984/119,919, 2%).

As the lockdown was implemented in France after public discussions and the official announcement by President Macron, *public measures* were more represented before the lockdown (12,014/34,327, 35%) than in the early lockdown period (16,788/119,919, 14%), and they were extensively discussed again with regard to the lifting of the lockdown during the mid to late lockdown period (127,334/282,965, 45%). Political criticisms and opinions were addressed in all three periods, as well as lockdown measures (the closing of nonessential locations during prelockdown, the strengthening of measures such as curfews in certain cities during early lockdown, and lockdown lift measures during mid to late lockdown). Specific criticisms of police actions, discussions of measures for the return to normal and the general impact of the lockdown (eg, economy and pollution), and debates regarding tracing apps were addressed during mid to late lockdown. People also shared comments about specific events such as the holding of mayoral elections that were scheduled just before the lockdown (including the preventive measures established) and the authorization of the prescription of hydroxychloroquine in hospitals.

Regarding *solidarity and encouragement*, most messages were about solidarity with frontline workers, especially in the context of mask and medical material shortages, and the importance of respecting preventive measures. Initiatives by organizations for citizens were publicized through Twitter over the three periods, such as regional news programs to inform people and organize solidarity, and initiatives to stay active personally and professionally. We also identified a topic related to free video content and media rivalries, as some media offered video content for free during the lockdown, but some of these actions were involved in copyright disputes and led to media rivalries.

### Comparison

The comparison of topics between Chinese and French social media is summarized in Figure 2. 

The common topics related to *epidemic news and everyday life* that were discussed in both countries included the evolution and statistics of the epidemic, everyday life, and personal difficulties and concerns. However, the concerns were not similar: Chinese users feared being infected and consequently asked for the lockdown of Wuhan, whereas French users were more concerned about the socioeconomic consequences and the enforceability of public measures. Similar topics were also addressed in regard to the *public measures* (eg, the chronology of measures) and *solidarity and encouragement* (eg, the importance of preventive measures and solidarity with health workers) categories. Notably, misinformation circulated through social media in both countries; however, it was more concerned with the virus and epidemic in China, whereas it was more concerned with the public measures before the lockdown announcement in France (eg, rumors about a general curfew for the whole country).

Moreover, we identified several dissimilar topics in all categories. The *epidemic news and everyday life* category included descriptions of early cases and situations in hospitals in China, in contrast to messages about shortages in supermarkets and criticisms of the behavior of the population in France. *Scientific information* was shared far more on Weibo in all three periods, covering virology, pathology, epidemiology, treatments, and vaccines, whereas in France, only one topic related to the virus and one topic related to hydroxychloroquine were identified at early stages. More criticisms were identified in French tweets than on Chinese Weibo in regard to the *public measures* category; such criticisms were directed toward the government, the political class, police, etc. Some country-specific subjects (eg, the day of mourning in China and mayoral elections in France) were also identified. News and discussions regarding tracing apps were addressed in French tweets; in addition to the advantages for controlling the epidemic after the lifting of the lockdown, people shared concerns about data privacy.

## Discussion

### Strengths and Limitations

Social media has demonstrated its value in helping people stay connected, delivering important public health messages, and disseminating important research findings in emergency situations. Building on our previous experience collating social media posts for pharmacovigilance monitoring [17], we present an analysis and comparison of the evolution of topics discussed on social media during the COVID-19 lockdown in China and France. Biterm topic modeling was used to identify topics in both corpora because of its superior performance on short texts from microblogging websites [18]. This automated approach enabled us to cluster and analyze the content of a data set that was much larger than in manual studies. Moreover, this comparative study was conducted by a multidisciplinary and bilingual team, the topics in each corpus were analyzed separately by two interpreters, and the final topics were assigned after alignment between the two countries and consensus of all interpreters. The qualitative analysis was completed through a rigorous two-step validation process. However, some limitations inherent to all studies using social media material remain. No medium has been proven to be representative of the general population; for example, only 56% of Chinese people use Weibo actively, and 34% of French internet users use Twitter actively [19]. In addition, there are some limitations related to the texts, such as the differences in the length of the messages on Twitter and Weibo and the presence of polysemic terms in the Chinese corpus.

### Interpretation of the Differences of Topics on Chinese and French Social Media

The use of social media has evolved rapidly and varies across different countries and cultural contexts. As one of the largest social media platforms in China, Weibo attracts not only ordinary users but also representatives from different sectors, such as celebrities, media, and government authorities. As of December 2019, there are 139,000 institutional government Weibo accounts, and public security agencies are the most representative government agencies on Weibo [20]. Moreover, as it dropped its 140-character limit in 2016, it has been largely used as an information source in recent years. Twitter is the most used microblogging platform in France; it still has a strict 280-character limit and has 34% of internet users aged 16-64 years as monthly active users [19]. In France, it is mainly considered a platform for sharing personal experiences and opinions. These different characteristics and cultural uses of Weibo and Twitter can partially explain the differences in the proportions of *scientific information* topics between China and France.

Another contributing factor is the different timelines of the evolution of the COVID-19 epidemic between December 2019 and May 2020. In late January, the prelockdown and early lockdown periods in China corresponded to the early stage of the new virus’s discovery, and less was known. When all European countries were affected by COVID-19 in early March, although they benefited from the knowledge and experiences acquired by Asian countries to control the epidemic, no treatment was then available. These elements could explain why some messages focusing on Dr Li Wenliang in China (who gave an alert about the first cases) and on Dr Didier Raoult in France (controversy over hydroxychloroquine) circulated.

The cultural context could also have an impact on the topics addressed. China is traditionally described as a country with a high tendency toward collectivism [21-23], which implies that harmony tends to prevail over personal opinions. In contrast, France has a strong culture of political debate, criticism, and opinion sharing. This tendency has already been observed on social media, as a previous study comparing the behavior of Twitter and Weibo users concluded that Weibo users tended to talk more positively about people than Twitter users [24]. These aspects may help to partly understand the different proportion of topics related to criticisms and debates regarding people’s behavior and politics. Moreover, the censorship exerted on social media in China [25] to comply with government requirements may have led to post deletions, which might have had an impact on the data collected and the topics addressed.

### Comparison With Prior Work

We searched MEDLINE via PubMed for evidence available by August 31, 2020, using combinations of the following terms: (“COVID-19” OR “lockdown”) AND (“social media” OR “Twitter” OR “Weibo”) AND (“topic model” OR “content analysis”). The search retrieved 9 relevant studies [26-34] among 20 published articles. We further identified 1 more relevant study [10] by screening bibliographies and “similar articles” suggested by PubMed.

Regarding the objective, most of the content analyses focused on a specific interest related to COVID-19, including public engagement and government responsiveness in Chinese Weibo during the early epidemic stage [26,27], nursing appeals on Twitter and Instagram in Brazil [28], pharmacists’ perception on social media in Jordan [29], the reason for not following the orders of the authorities [30], the self-reported symptoms [31], and the misinformation about a particular subject, like 5G spreading COVID-19 in the United Kingdom [32] or general misinformation fueled by rumors, stigma, and conspiracy theories [33]. In these cases, the identified content patterns or topics were related to the specific interest. For example, in the self-reported symptoms–related study [31], the identified topics included reports of symptoms, lack of testing, recovery discussion, and negative diagnosis. There are only 2 studies with general interest on COVID-19–related topics, 1 on Chinese Weibo [34] and the other on English Twitter [10].

For the design and settings, most research efforts have been devoted to the early stage of the epidemic [26,27,34] or focused on a short period of 2 or 3 weeks [28-32], without a particular interest in the evolution of the topics addressed during the lockdown. Most of these prior studies considered messages in one social media platform or in one language, most often English Twitter followed by Chinese Weibo, except for the general misinformation-related study [33], which identified 2311 reports of rumors, stigma, and conspiracy theories in 25 languages from 87 countries.

With regard to the method, except for the general topic study [10] that used the latent Dirichlet allocation model and the self-reported symptom–related study that used the biterm topic model [31] to automatically group social media posts into topic clusters, most of the other studies performed a manual content analysis, which limited the size of the data set.

In terms of the results, the topics discussed in Chinese Weibo were similarly identified in prior studies [26,34], which are comparable to our results on Chinese Weibo during the prelockdown and early lockdown periods. Our results even covered many of the topics identified in those studies with a specific interest. For example, in the nursing appeals–related study [28], the emerged thematic categories of #stayathome and #whereismyPPE were also identified through our analysis and overlapped with some topics in the *solidarity and encouragement* category. Another example, the study exploring why people ignore the orders of the authorities [30] revealed reasons such as information pollution on social media, the persistence of uncertainty about the rapidly spreading virus, the impact of the social environment on the individual, and fear of unemployment. All these aspects were addressed in our results. The public engagement and government responsiveness discussed by Liao et al [26] and the significance of regulating misinformation highlighted by Ahmed et al [32] and Islam et al [33] also emerged in either Chinese topics, French topics, or both through our analysis. Comparing with the general topic study on English tweets [10], which identified topics and determined the sentiment (positive or negative) for each topic, although the mean sentiment was positive during the period between early February and mid-March 2020 (10 topics out of 12), 2 topics conveyed specific concerns about the deaths caused by COVID-19 and increased racism. Contrasting with these findings, we did not identify any topic about racism in Chinese Weibo nor in the French messages. In China, instead of racism, regional discrimination against people from Wuhan was discussed in several Weibo messages (eg, some hotels refused to accept guests from Wuhan). These messages were clustered into the topic related to personal difficulties during the lockdown. In French social media, tweets related to group conflict were more about discriminatory actions by the police against homeless people, criticisms of privileged people who left big cities for their secondary house and may have spread the epidemic in preserved regions (in particular criticism of the Parisians who left Paris at the beginning of the lockdown), and criticisms of people from deprived neighborhoods (sometimes described as people with immigrant backgrounds) for not respecting the lockdown. These messages were clustered into other topics, like “criticism of police actions” and “non-observance of preventive measures, irresponsibility of the French.”

### Implications for Public Health Policies and Perspectives

In contrast to the expected value of social media in sharing information about the epidemic and assessing public needs and opinions in emergency situations, our study has also identified several common risks of Chinese and French social media. The main risk is the widespread sharing of fake news (eg, regarding the virus and public measures), which can lead to mistrust, fear, and improper actions. Even premature findings that have not yet been validated but that spread quickly and widely on social media can cause problems, such as self-medication with unproven treatments (efficacy and safety) and drug shortages (Shuanghuanglian in China and hydroxychloroquine in France). Moreover, scientific information conveyed to the public in an inappropriate way can lead to misunderstandings and unnecessary panic (eg, transmission via aerosols in China). These common risks highlight the importance for institutions to establish an effective communication system to provide reliable, appropriate, and understandable information.

Moreover, our study revealed several different concerns expressed by the public through Weibo and Twitter that may be addressed by Chinese and French policy makers. In China, worries about this new virus and disease, combined with fears of being infected, led to numerous requests for the lockdown of Wuhan on Weibo before the lockdown was implemented. As a result, the lockdown decision was welcomed by many Weibo users in China. In contrast, in France, worries about socioeconomic consequences, impacts on everyday life, and concerns about food shortages in supermarkets were frequently expressed in French tweets. Regarding scientific information, the lack of established knowledge about the new virus and disease led to two different types of messages: the sharing of scientific information among Weibo users, on the one hand, and opinions and controversies about treatment and hydroxychloroquine in France, on the other hand. Ultimately, concerns regarding tracing apps and data privacy were identified in the French tweets, underlining the need for institutions to provide a clear regulatory framework and appropriate messages to the population so that these technologies are trusted and widely spread. These different concerns suggest country-specific needs that should be sufficiently addressed by decision makers.

## Appendix

Multimedia Appendix 1 Keywords for extraction.

Multimedia Appendix 2 Preprocessing.

Multimedia Appendix 3 Chinese topics in the prelockdown period.

Multimedia Appendix 4 Chinese topics in the early lockdown period.

Multimedia Appendix 5 Chinese topics in the mid to late lockdown period.

Multimedia Appendix 6 French topics in the prelockdown period.

Multimedia Appendix 7 French topics in the early lockdown period.

Multimedia Appendix 8 French topics in the mid to late lockdown period.

## Abbreviations

API: application programming interface
